# Strategic Withdrawal as Empowerment for Individuals With Depression in Internet Support Groups: Qualitative Study

**DOI:** 10.2196/92690

**Published:** 2026-08-14

**Authors:** Yufan Zhang, Zhentao Liang

**Affiliations:** 1 School of Journalism & Communication, Chongqing University, Chongqing, China; 2 School of Information Management, Wuhan University, 299 Bayi Road, Wuhan, Hubei, 430072, China, +86 (027) 68752135

**Keywords:** depression, mental health recovery, internet support group, withdrawal, empowerment, social support, peer support, self-management, digital health, eHealth, disengagement, qualitative research

## Abstract

**Background:**

Internet support groups (ISGs) provide accessible peer support for individuals with depression; yet, their withdrawal rates are substantially higher than those observed in general online groups. Existing research has focused primarily on retention as a marker of intervention success. The significance and mechanisms of withdrawal, therefore, remain underexplored.

**Objective:**

This study aimed to examine how individuals with depression use withdrawal from ISGs as a strategy for empowerment. It also sought to clarify how autonomy, courage, and responsibility are enacted through the process of leaving.

**Methods:**

We conducted a qualitative study using semistructured interviews with 19 participants. They were recruited through purposive and snowball sampling on a major Chinese social media platform. The sample comprised 18 former ISG members diagnosed with depression and 1 in-group psychological counselor. Among the former members, 5 had served as group founders or administrators. Data were collected through audio-, video-, and text-based interviews and were subsequently transcribed and deidentified. Analysis followed a hybrid deductive and inductive approach. A priori categories drawn from the empowerment framework were treated as provisional and remained open to revision based on the data. In parallel, inductive grounded theory procedures identified emergent patterns of strategic withdrawal. Negative case analysis, blind coding, external expert review, and member checking were used to strengthen rigor.

**Results:**

In participants’ accounts, withdrawal from ISGs emerged as a multidimensional empowerment process rather than a single act. Autonomy grounded the process. Participants reassessed the gap between their expectations and the group reality and came to judge their recovery by their own standards. Courage carried that judgment into action. Whether through decisive exits or gradual disengagement, participants broke the inertia of habitual connection and re-established personal boundaries. Responsibility did not wait for the exit. It surfaced beforehand as a felt obligation to protect one’s own health, which helped drive the decision to leave. After withdrawal, it matured into sustained self-management as participants redirected their energy toward offline recovery and alternative sources of support. The 3 dimensions did not form a linear sequence. Instead, they unfolded in a staggered and interwoven manner throughout the process. Negative cases, including externally imposed exits and returns driven by fluctuating symptoms, delimited the scope of these findings.

**Conclusions:**

This study challenges the retention-focused perspective in ISG research by showing that strategic withdrawal can be an empowering process for individuals with depression. Empowerment is achieved not only through ongoing participation but also through the autonomy to disconnect, the courage to set boundaries, and the responsibility to manage one’s own recovery. Mental health professionals and platform designers should recognize and support strategic withdrawal as a legitimate and positive outcome. Such recognition would help foster a more resilient and patient-centered digital support ecosystem.

## Introduction

The increasing accessibility of digital platforms [1], combined with challenges in obtaining offline mental health support [2], has led to the rapid proliferation of internet support groups (ISGs). These groups provide safe and accessible spaces for a wide range of interpersonal exchanges and social learning. Many participants report that such opportunities help them better accept and manage their illness while also increasing confidence in their own strength [3]. Empowerment is frequently used as an explanatory concept in ISG research to capture this type of change [2-4].

Empowerment is broadly understood as a process concerned with individuals’ determination over their own lives [5]. It also functions as an open-ended organizing construct that does not specify what a person or setting is empowered to do [5]. This openness has been accompanied by varied operationalizations across studies, including differences in level of analysis [6], process versus outcome assumptions [7,8], and trait versus state assumptions [6]. Nevertheless, several recurring themes can be identified across this literature. Across empowerment research that has informed health and mental health contexts, recurring themes include gaining personal control, reducing powerlessness, and developing the capacity to act on one’s circumstances. Zimmerman’s [6] community psychology model, for instance, conceptualizes psychological empowerment through intrapersonal, interactional, and behavioral components. It links perceived control and self-efficacy to an understanding of one’s environment and to action [6]. Similarly, the consumer-constructed empowerment scale in mental health services emphasizes self-esteem and self-efficacy, reduced powerlessness, community activism, autonomy, optimism, and control over the future, and righteous anger [9].

Within general medical literature, empowerment has been conceptualized as arising from communication and shared decision-making between health care professionals and patients, centering on the dynamics of that relationship [7,8]. In contrast, in the mental health recovery literature, empowerment is framed as a corrective to the dependency that can develop from prolonged reliance on a system of care [10]. This framing centers on the individual’s own trajectory rather than on a specific clinical relationship. Building on this recovery-specific framing, Jacobson and Greenley [10] operationalize empowerment for individuals in recovery through 3 interrelated components. Autonomy refers to the capacity to act independently. Courage involves the willingness to take risks, speak authentically, and step beyond familiar routines. Responsibility denotes the felt obligation to care for oneself. These components correspond to the recurring themes identified above. Rather than treating empowerment only as a static perception of control or self-efficacy, this framework conceptualizes it as a process of moving from dependency on external sources of support toward greater self-directed living [10].

People with depression are among the most active ISG users. They seek to activate these dimensions by sharing experiences, gaining peer support, and learning coping strategies [3,11-13]. However, a paradox emerges between this active pursuit of empowerment and actual patterns of user engagement. Although ISGs have been shown to improve self-efficacy [14-16] and deliver clinical benefits [17,18], they also experience exceptionally high attrition rates. Nearly half of participants disengage within 6 months of joining [19]. This contrast raises a fundamental question: “Does leaving an ISG represent a failure of the intervention, or does it signal that users have achieved the empowerment they were seeking?”

This question becomes even more urgent given the stark difference in retention rates. While general online groups typically see an attrition rate of just 13.1% over a comparable period [20], ISGs have a withdrawal rate of nearly 50% [19]. In response, existing research has focused on strategies to improve user retention, operating under the assumption that ongoing engagement is essential for empowerment [13,21]. Studies have demonstrated that the online environment provides crucial scaffolding for users to activate empowerment. Peer support enables them to learn coping strategies and manage recovery (responsibility) [3,22], while group anonymity encourages self-expression and sharing (courage) [3,11,23]. As a result, continuous engagement is often seen as a prerequisite for these benefits. Social network embeddedness [19,24] and emotional belonging [21,25] have been identified as key factors in preventing exit. However, this retention-focused perspective overlooks a critical issue. By equating empowerment with engagement, it suggests that leaving entails a loss of these benefits [13]. This view implicitly casts individuals as passive recipients who depend on constant connectivity for well-being, neglecting the critical dimension of autonomy, the ability to act independently [10]. While sustained engagement may foster responsibility and courage through external support, it can also undermine autonomy by promoting “digital dependency” or worsening depressive symptoms through excessive connectivity [26-29]. Therefore, the high attrition rate should not be viewed simply as a failure to maintain support. Instead, it may reflect a complex trade-off, with users relinquishing external benefits to reclaim the autonomy needed for independent living.

Some research has examined the potential causes of attrition, often pointing to internal issues within ISGs as key drivers. For example, information overload can result in cognitive fatigue and drown out meaningful support, leaving participants’ needs unmet [3,11,30]. Furthermore, the prevalence of emotional contagion and negative comparison in ISGs for people with depression can lead to social withdrawal and exacerbate self-stigma [1,3,11,23,31,32]. In such cases, the tendency of positive-oriented users to leave ISGs [19] is frequently interpreted as a reactive response to a toxic environment. However, this interpretation overlooks the proactive nature of withdrawal in an era characterized by being “permanently online, permanently connected” [33]. Current debates often frame disconnection as a passive, frustrated retreat [34,35], failing to recognize that initiating withdrawal requires conscious agency to prioritize personal boundaries over social expectations. Practices such as “digital detoxes” [36] or limiting connectivity [37,38] are deliberate strategies aimed at restoring a sense of control [39,40]. Viewed from this perspective, exiting is not merely an escape from stress but a purposeful act of empowerment. Specifically, it reflects the autonomy to self-regulate, which is fundamental to the recovery process [10].

However, empirical evidence supporting withdrawal as a positive outcome remains limited, as existing studies predominantly rely on data from members who remain engaged [3,41,42]. Without insights from those who have left, the specific mechanisms through which withdrawal operates as an empowerment strategy remain unclear. To address this gap, we focus on individuals with depression who have voluntarily discontinued their participation in ISGs. Through qualitative inquiry, we aim to elucidate how participants use withdrawal as a strategy to reclaim personal boundaries, examining how autonomy, courage, and responsibility are enacted in this process. By centering the experiences of those who leave, this research reframes withdrawal not as the end of care but as a critical, autonomous milestone in the journey of recovery.

## Methods

### Research Design

In this qualitative study, we conducted semistructured interviews with individuals diagnosed with depression who had voluntarily disengaged from ISGs, supplemented by an interview with an in-group psychological counselor. We aimed to investigate how withdrawal functions as a form of empowerment. To address the complexity of this phenomenon, we adopted a hybrid analytical approach that integrated both deductive and inductive reasoning [43]. This methodology enabled us to construct an initial coding framework rooted in empowerment theory [10] while also allowing us to remain receptive to emergent insights from the data [44]. By combining theoretical rigor with contextual sensitivity, our approach ensured a nuanced understanding of the withdrawal process.

Our deductive analysis was based on the empowerment framework [10], which comprises the core dimensions of autonomy, courage, and responsibility. To translate these psychological constructs into concrete analytical categories, we operationalized them as evaluation (assessing the group’s utility and harm), disconnection (specific behaviors that reduce or sever contact), and self-management (adopting alternative recovery methods). We initially assumed a broad correspondence between each dimension and one phase of the withdrawal process. We treated these categories as a provisional starting point rather than hypotheses to be confirmed. They allowed us to examine the abstract concept of empowerment within the real-world context of ISGs for people with depression while leaving the framework itself open to revision based on the data. This assumed correspondence was, in fact, substantially revised during analysis.

Next, using the inductive, data-driven coding procedures of grounded theory [44,45], we identified context-specific patterns of proactive disconnection. To minimize potential bias from theoretical assumptions, we adopted a 3-stage coding process consisting of open, axial, and selective coding [44,46]. This iterative approach continued until theoretical saturation was achieved, defined as the point at which additional interviews no longer yielded new themes related to withdrawal mechanisms or the expansion of empowerment.

### Study Participants

Nineteen participants were recruited through a 4-stage purposive sampling process on RedNote (Xingyin Information Technology (Shanghai) Co, Ltd) between November 22, 2025, and January 15, 2026. RedNote, a major Chinese social media platform with over 300 million users, serves as a prominent hub for mental health discussions, with depression-related topics amassing more than 3.3 billion views. Potential candidates were identified by filtering discussion threads using keywords including “depression groups” (抑郁群), “psychological mutual aid” (互助), and withdrawal-related terms such as “quitting the group” (退群). Through purposive sampling [47] and snowball sampling [48], we assembled a participant pool that captured a range of motives for leaving, as well as diverse forms of empowerment across varying engagement backgrounds.

The first stage aimed to construct a diverse patient sample through preliminary screening. Eligible patients were required to meet three inclusion criteria: (1) have a formal diagnosis of depression and be in a stable emotional state, (2) have continuously participated in an ISG for more than 2 weeks, and (3) have voluntarily withdrawn, muted, or stopped checking the group for at least 2 weeks. Withdrawal was defined to include explicit exit from the group, muting notifications, and ceasing to check the group, each sustained for at least 2 weeks. Treating these behaviorally distinct forms under a single definition was an analytic decision grounded in the composition of the sample and early analysis. The 3 forms did not mark separate participant subgroups. Of the 18 patients, 17 explicitly exited at least one group, and muting frequently functioned as an intermediate step, with 4 patients first muting a group before subsequently exiting it. Interview data further indicated that the 3 forms expressed a single underlying process of intentionally withdrawing attention and investment. They differed in degree rather than in kind, ranging from abrupt exit after acute conflict (eg, P4), through gradual fading (eg, P6), to periods of disengagement without the behavioral marker of exit (eg, P15). This continuum is discussed in greater detail in the Results section. P13 was the only patient who never actively exited a group. She described herself as someone who did not typically leave groups; her disengagement followed a mixed pathway. Several of her groups were dissolved by the platform for suicide-related content. We analyzed these externally imposed endings separately because they fall outside strategic withdrawal (see Analysis Strategy). By contrast, she voluntarily muted and stopped checking her remaining groups once she no longer felt a need for them. This voluntary disengagement qualified her for inclusion. Before the interviews, direct messages were sent to introduce the study background, objectives, and anonymity protocols. This stage concluded with 6 participants representing a range of ages, backgrounds, and group experiences.

The 2-week threshold was not fixed a priori. Recruitment initially targeted participants with at least 6 months of engagement, following the median active period reported for online mental health communities by Wang et al [19]. The threshold was lowered during fieldwork after early interviews revealed brief but highly consequential group experiences. For example, P4 joined another group for only 2 days, yet the experience left a lasting impression on her. The decision was also informed by the counselor’s observation that members whose needs go unmet may withdraw quickly but with clear evaluative reasoning. Participation duration in the final sample ranged from 2 weeks to 6 years. Eleven patients had been engaged for 6 months or longer, and only 2 for less than 1 month. The 2-week case (P8) was retained after analysis showed that depth of engagement, rather than duration alone, determined the reflectiveness of the withdrawal process. Such rapid yet reflective withdrawal appeared to require unusually intense involvement. For most participants, however, a longer period of engagement remained the more common pathway to evaluative withdrawal.

The second stage incorporated the perspectives of group administrators and professionals to enrich the core concepts with structural and expert insights. Initial analysis of the first round of interviews indicated that individual perspectives alone were insufficient to fully capture the dynamic role of ISGs in the empowerment process. Therefore, we recruited 6 participants with specialized roles, including 2 ISG founders, 3 administrators, and 1 in-group psychological counselor (C19). C19 was not a patient but participated in the ISG in a facilitative capacity and provided a professional perspective on group processes. This approach enabled cross-validation of findings from managerial and professional viewpoints. Notably, the founders and administrators recruited at this stage were themselves patients with a formal diagnosis of depression. This stage was designed in part to examine whether their structurally distinct positions within the group, such as holding authority over group norms or greater awareness of member attrition, produced a withdrawal experience different from that of regular members.

Our analysis indicated that, while an administrative dimension was sometimes present, participants’ withdrawal narratives remained anchored in their identities and needs as patients. For the founder of a small, friendship-based group (P11), withdrawal was described in terms indistinguishable from a regular member leaving a social circle. Even for the founder of a larger, stranger-based group (P10), whose group gradually fell silent, the stated reason for leaving was an unmet patient-level need to discuss treatment with others facing the same condition rather than a managerial responsibility to sustain the group. The 3 administrators showed the same pattern. The informational support they occasionally provided to other members was framed as part of managing and documenting their own treatment (eg, P3). Their exits were likewise driven by patient-level evaluations of the group environment rather than role-based obligations. We therefore treated the administrative role as a contextual feature of the withdrawal experience rather than a basis for separate analytical treatment because our analysis centers on patient identity rather than in-group role.

The third stage aimed to challenge and refine the analysis by including negative cases and atypical samples. To enhance theoretical reliability and reduce confirmation bias, we recruited participants whose experiences diverged from mainstream narratives. This included participants who left despite holding highly positive views of the ISG and users who frequently returned after exiting. Comparing these divergent perspectives allowed for a more rigorous understanding of empowerment. Recruitment continued through the fourth and final stage of supplementary interviews until theoretical saturation was achieved. The final sample consisted of 19 participants, including 18 patients and 1 nonpatient counselor (C19).

Table 1 provides an overview of the demographic characteristics, medical histories, and ISG engagement patterns of all participants (N=19), including 18 patients and 1 nonpatient counselor (C19). C19 was included in the full sample (N=19) but was excluded in the quantitative summaries of patient characteristics. C19 is reported separately due to their distinct professional role within the ISG. The sample included individuals with formal clinical diagnoses spanning onset dates from 1994 to 2024. This ensured coverage of various stages of recovery, from long-term management to recent diagnosis. ISG participation ranged from 2 weeks among newer members to 6 years among core participants. This diversity of roles and withdrawal durations offers a robust foundation for multidimensional analysis of the dynamic logic underlying ISG exit, which is elaborated in the Results section.

**Table 1.** Demographic and clinical characteristics of participants (N=19), including patients (n=18) and 1 nonpatient counselor (C19; n=1), and their ISG^a^ engagement patterns^b^.

Characteristics	Participants (N=19)	
	Patients (n=18)	Counselor (n=1)
Demographic characteristics		
Age (years), median (range)^c^	26 (18‐48)	34
Sex, n (%)		
Female	13 (72)	0 (0)
Male	4 (22)	1 (100)
Refuse answer	1 (6)	0 (0)
Education, n (%)		
High school	5 (28)	0 (0)
Associate degree	4 (22)	0 (0)
Bachelor’s degree	6 (33)	1 (100)
Master’s degree	1 (6)	0 (0)
Refuse answer	2 (11)	0 (0)
Occupation, n (%)		
Health care/counseling	1 (6)	1 (100)
Other employed	11 (61)	0 (0)
Unemployed	4 (22)	0 (0)
Student	1 (6)	0 (0)
Refuse answer	1 (6)	0 (0)
Clinical characteristics		
Time since diagnosis (years), n (%)		
1‐3	3 (17)	—^d^
4‐9	13 (72)	—
≥10	2 (11)	—
Sought professional help before ISG, n (%)		
Yes	17 (94)	—
No	1 (6)	—
Current treatment status, n (%)		
Ongoing treatment	14 (78)	—
Recovery	4 (22)	—
ISG participation		
Time in ISG^e^, n (%)		
2 weeks-2 months	4 (22)	1 (100)
2 months-6 months	3 (17)	0 (0)
6 months-1 year	2 (11)	0 (0)
1‐3 years	5 (28)	0 (0)
≥3 years	4 (22)	0 (0)
Time since leaving, n (%)		
<1 year	6 (33)	1 (100)
1‐3 years	8 (44)	0 (0)
≥3 years	4 (22)	0 (0)
ISG role, n (%)		
Founder/administrator	5 (28)	0 (0)
Regular member	13 (72)	0 (0)
Professional facilitator	0 (0)	1 (100)

a ISG: internet support group.

b The percentage values in this table may not be equal to 100% owing to rounding.

c Values are reported as median (range), not median (IQR). For the patients, the median age was 26 years, with a range of 18-48 years. As there was only 1 counselor, the counselor’s age is presented as 34 years, without a range.

d Not applicable.

e Duration bands are left-closed and right-open intervals. For example, the band from 6 months to 1 year includes durations of exactly 6 months and excludes 1 year, which falls in the next band, and the band of 3 years or more includes durations of exactly 3 years.

### Data Collection

A preliminary interview guide was developed following a broader review of the literature on online support groups. Two studies were identified as being most directly relevant to our research questions. Smit et al [3] inquired about participants’ motivations for joining an ISG and their participation patterns, shaping the guide’s first 2 domains (motivations for joining the ISG and experiences during participation). Wang et al [19] informed our understanding of the withdrawal process itself, shaping questions on participants’ thinking at the time of withdrawal and whether they sought alternative forms of support afterward. These questions formed the guide’s remaining 2 domains (mechanisms of leaving and postwithdrawal recovery). Exploratory discussions to draft this preliminary guide were held between the 2 authors (YZ and ZL) responsible for its development.

Before the formal study, this preliminary guide was piloted with a psychiatrist from a tertiary care hospital and a former ISG participant with approximately 7 months of group experience. Based on their feedback, we made several refinements to the guide. These included adding a question about whether participants were already receiving treatment prior to joining the group to better clarify their underlying motivation, broadening the scope of participation-related questions to capture negative as well as positive experiences, and removing a few items that overlapped with others already covered. The guide was then finalized through iterative discussions among the authors and subject matter experts. The final version encompassed four core domains: (1) motivations for joining the ISG, (2) experiences during participation, (3) mechanisms of leaving, and (4) postwithdrawal recovery. The complete interview guide is provided in Multimedia Appendix 1, and its core domains are summarized in Textbox 1.

Textbox 1. **Themes and subthemes in the interview guide.**

- Motivations for joining the internet support group (ISG)
  - Motivations, channels for joining, and other forms of help sought before joining
  - Initial expectations and first impressions upon joining
- Experiences during participation
  - Participation styles and perceived support
  - Experiences and interpersonal conflict
- Mechanisms of leaving
  - Triggers and psychological turning points for withdrawal
  - Decision-making process and self-interpretation of the exit
- Postwithdrawal recovery
  - Reconfiguration of offline support networks
  - Self-assessment of emotional and social functioning postexit

After the initial 6 interviews, previously unexplored themes were reviewed, and additional questions were incorporated to further probe participants’ perceptions of their withdrawal [49]. Audio and video interviews were recorded with informed consent and transcribed verbatim. Text-based records were exported directly as raw data. To ensure confidentiality, all data were rigorously deidentified by removing personally identifiable information and replacing participant names with alphanumeric codes (eg, P1 and P2 for patients and C19 for the counselor).

Between November 22, 2025, and January 15, 2026, all interviews were conducted by the first author (YZ), who has a nursing background and has received systematic training in qualitative research methods. To manage the potential influence of this clinical perspective, the interviewer maintained reflexive notes after each session to remain focused on participants’ personal narratives. One-on-one interviews were chosen to foster rapport and maintain stable interaction with participants, which is crucial when addressing sensitive topics such as motivations for withdrawing from ISGs [50]. To minimize emotional burden and enhance participants’ sense of security, several interview formats were offered according to individual preference, including face-to-face, video calls, audio calls, or text-based asynchronous interviews. Interview durations varied according to communication needs, with oral interviews lasting from 60 to 150 minutes and text-based sessions spanning from 2 hours to 5 days. Emotional support was provided before and after each session, and the interviewer remained available for ongoing communication throughout the study.

### Analysis Strategy

After deidentification, all interview transcripts were imported into NVivo 15 (Lumivero LLC) and analyzed using a hybrid deductive and inductive approach [43]. In the deductive phase, we established a priori thematic codes based on the empowerment framework [10] and our operationalized categories of evaluation, disconnection, and self-management, onto which the raw data were initially mapped as a starting point [51]. These codes were treated as provisional sensitizing concepts rather than hypotheses to be confirmed [52]. We therefore remained attentive to inductive codes that could not be mapped onto any predefined category, codes that recurrently spanned categories, and negative cases that contradicted category assumptions. The third sampling stage, which recruited participants who repeatedly returned after leaving and those who left despite positive views of their ISG, was designed to surface such potentially disconfirming accounts.

To avoid theoretical forcing and remain sensitive to emergent patterns [53], 2 authors (YZ and ZL) conducted open, axial, and selective coding [46,54]. This process involved line-by-line open coding to generate emergent labels and axial coding to link these subthemes to the empowerment dimensions. This iterative process continued until additional interviews yielded no new themes concerning withdrawal mechanisms.

This procedure led to 2 substantive revisions of the deductive structure. First, codes describing participants’ concern about protecting their own well-being, initially generated under evaluation, overlapped substantially with codes independently developed under self-management. We treated this overlap as evidence against the assumed one-to-one mapping between dimensions and process phases and instead traced the form each dimension took across the process, yielding the temporal structure reported in the Results. Each dimension was most salient in one phase yet could recur across phases in different forms and degrees of maturity. Responsibility, in particular, appeared before withdrawal as a felt obligation to protect one’s own health that helped drive the decision to leave and after withdrawal as enacted self-management. The source framework presents the 3 dimensions as parallel components and specifies no such temporal patterning [10].

Second, negative case analysis delimited the scope of the empowerment reading. Exits imposed from outside, such as groups dissolved through platform moderation, involved no exercise of choice and fell outside the core category of strategic withdrawal. This category was itself generated inductively because the source framework contained no concept of disconnection. Other accounts qualified the interpretation rather than exemplified empowerment. Some participants cycled between joining and leaving as their condition fluctuated and described their decisions as provisional, indicating that the dimensions could be enacted in unstable and reversible ways. Others attributed their recovery mainly to treatment and self-directed effort and regarded the exit itself as inconsequential, cautioning against overstating the causal weight of withdrawal. These cases are reported in the Results as boundary conditions. Throughout this process, the authors held regular reflexive sessions to challenge their own assumptions while organizing the 94,700-word corpus. They paid particular attention to whether negative cases were adequately represented rather than explained away.

To ensure the rigor and validity of the analysis, several verification strategies were used. First, the authors independently performed blind coding on portions of the data and met at each stage to discuss discrepancies. Inconsistent codes were refined into smaller units and redefined until consensus was reached. Second, the preliminary themes were shared with 3 external experts who were not involved in the project. Their external perspectives allowed us to scrutinize the logical consistency of the coding framework and thematic interpretations. Finally, to enhance credibility and mitigate researcher bias, we conducted member checking by sharing the synthesized findings (ie, core themes) with 7 randomly selected participants. They were invited to verify whether the interpretations accurately reflected their lived experiences [55].

### Ethical Considerations

This study was conducted in accordance with the Declaration of Helsinki and all relevant institutional and national ethical guidelines. The research protocol was reviewed and approved by the Wuhan University Ethics Committee on November 10, 2025 (approval number WHU-HSS-IRB2025095). Before participation, all individuals were fully informed about the study objectives, procedures, potential risks and benefits, their right to withdraw at any time without penalty, and the measures taken to ensure data protection and confidentiality. Informed consent was obtained from each participant, either in writing or orally, depending on the interview format. To protect privacy, all personally identifiable information was removed from the transcripts, and participants were assigned alphanumeric codes (eg, P1 and P2) for data analysis and reporting. As compensation for their time and contributions, each participant received RMB 60 (approximately US $8.61; US $1=RMB 6.9660 as of January 15, 2026) or an equivalent small gift upon completion of the interview.

## Results

### Overview

Interview data indicate that, for individuals with depression, withdrawing from an ISG can constitute a multidimensional empowerment process. The findings are organized into 3 sections corresponding to the core dimensions of empowerment, each of which was most salient during one phase of the withdrawal process. The section on autonomy examines how participants weighed group dynamics against their own needs while still in the group and formed an independent judgment about leaving. The section on courage centers on the act of disconnection itself and examines how participants described breaking the inertia of connection and prioritizing personal boundaries. The section on responsibility examines how, after withdrawal, participants took over management of their own recovery.

The 3 dimensions did not unfold in strict sequence. Autonomy and courage retained a stable form wherever they appeared, whereas responsibility matured over the course of the process. It surfaced well before exit as a felt obligation to protect one’s own health, which participants described as part of what drove the decision to leave, and was enacted as active self-management only after withdrawal.

### Autonomy: Reclaiming Independent Judgment

#### Overview

Individuals’ evaluation of ISGs shifted from initial expectations toward a more realistic assessment. This change is driven by the discrepancy between anticipated support and the reality of group dysfunction. As individuals engaged with ISGs, their criteria for evaluating recovery gradually shifted. Initially, they relied on group norms or the experiences and judgments of others, seeking validation and support from the collective. Over time, this orientation gave way to a more introspective approach. Individuals began to assess their own experiences and recovery needs according to personal standards and self-reflection. This transition from external reference to internal judgment reflects what the empowerment framework describes as acting as an independent agent, drawing on the tools of autonomy it identifies, namely, knowledge, self-confidence, and the availability of meaningful choices [10].

Among the 18 patients, most joined ISGs to seek emotional or informational support, motivated by a desire for empathy and improved disease management. These initial expectations were often shaped by an “oasis illusion” (C19), in which the group was seen as a potential refuge. For instance, P13 shared:

I joined the group hoping for even the slightest chance of further healing, especially when conventional treatments like medication and counseling offered limited relief.

[P13, female, 22 years]

However, as the gap between expectations and reality became clear, participants’ accounts traced a shift from passive recipients of support to active evaluators of the group. They began to recognize factors within the group environment that they experienced as hindering or even harming their recovery. The decision to leave thus grew out of this renewed exercise of independent judgment. This process involves 3 themes: recognizing risks within the ISG environment, de-idealizing the functions of ISGs, and making strategic decisions to withdraw, a step in which an emerging sense of obligation to their own health also came into play.

#### Recognizing Risks Within the ISG Environment

As their engagement in the ISG deepened, many participants reported experiencing negative effects of the group. Rather than offering the expected support, the group often became a source of stress according to participants’ accounts. Frequent displays of negativity, such as self-harm rhetoric and competitive venting, caused considerable distress. Through ongoing interaction, individuals described gradually learning to identify and navigate these environmental risks.

The group was nothing like what I had anticipated. It included not only people with depression but also those struggling with anxiety, schizophrenia, and bipolar disorder. Members would impulsively vent their emotions daily, and it felt as though everyone was isolated in their own world, with little effort to understand others’ experiences. Conflicts among members were frequent, and the overall negative atmosphere became overwhelming, leaving me feeling increasingly irritable.

[P8, female, 36 years]

Recognizing these risks marked a growth in autonomy. What changed in these accounts was less the group itself than participants’ knowledge of the group. This knowledge led them to feel that safeguarding their own well-being mattered more than maintaining ties with the ISG. Both P9 and P18 described distancing themselves as essential for psychological self-protection when the stress generated by the group surpassed what they felt able to cope with.

The constant occurrence of extreme situations in the ISG heightened my stress, prompting me to seriously consider leaving the group altogether.

[P9, female, 36 years]

Individuals further exercised autonomy by critically evaluating the effectiveness of the ISG. Their assessment went beyond simply recognizing negative emotions. They questioned whether the group genuinely provided meaningful support. Some participants joined ISGs expecting constructive information exchange and professional guidance, only to find a lack of substantive interaction. As P16 noted, an unresponsive ISG failed to meet her need for genuine interpersonal connection.

The group felt really quiet. It didn’t seem to matter whether I was there or not. I was very active when I first joined, but hardly anyone posted questions or opened up about their feelings.

[P16, female, 25 years]

She also highlighted the shortcomings of ISGs that claimed professional expertise, suggesting that her evaluation was grounded in the quality of interactions rather than mere credentials or authority.

There were 2 counselors and a psychiatrist in the ISG who would post educational materials, but no one ever interacted with them. Sometimes they sent holiday greetings, but even when the group founder mentioned them, they never responded to participants. I really couldn’t figure out what their role was in the group.

[P16, female, 25 years]

#### Deidealizing the Functions of ISGs

Many individuals no longer regarded ISGs as consistently effective avenues for recovery. This shift reflects a growing sense of autonomy, grounded in the knowledge of the group that participants had accumulated. Rather than relying on the group for solutions, participants started to chart their own paths to recovery by acknowledging the limitations of ISGs.

First, participants described a lack of professionalism in the information shared within ISGs. Groups that primarily serve as outlets for emotional venting often lack clinical guidance, compelling participants to rely on their own judgment to filter out misinformation. Rather than accepting advice uncritically, they evaluated the content carefully to avoid potentially harmful recommendations.

The help I received was minimal, although I did pick up some information about policies and medications. In fact, I often found myself providing science outreach to others because very few people were actually well-informed. There was a lot of misinformation; some even pretended to be doctors and suggested things like prefrontal lobotomies.

[P3, female, 22 years, internet support group (ISG) administrator]

Second, participants felt that heterogeneity among members could hinder genuine empathy. Differences in age and life circumstances often made it difficult for peers to truly understand one another. Coming to feel that a shared diagnosis did not necessarily lead to shared understanding, they began to prioritize their own needs over a generalized sense of group belonging.

Just because we share the same illness doesn’t mean we truly understand each other. Honestly, sometimes I don’t even understand myself. Everyone’s situation is different, and some people even mock or criticize others.

[P1, male, 48 years]

Most of the people in ISGs are quite young. They’re focused on family, school, or the challenges of starting out in society. They don’t really understand or empathize with the pressures I face as someone in middle age, and I find their casual words of comfort unhelpful.

[P9, female, 36 years]

Finally, participants described the support of ISGs as limited in duration. They observed that while a group may be beneficial at first, it can become less valuable and even a source of stress as their condition evolves. This perception leads them to view the ISG as a temporary resource rather than a lasting solution.

If you stay too long, it’s bound to turn into a negative experience. I prefer to leave as soon as I sense things change, so I can keep the good memories and avoid unnecessary disappointment. The impact of an ISG isn’t constant. Once the ‘honeymoon period’ is over, I’d rather leave than collect more unpleasant memories.

[P11, female, 25 years, ISG founder]

#### Making Strategic Decisions to Withdraw

Once they perceived the ISG as risky and of limited value, participants moved from contemplation to action and made a decisive choice to leave. In their accounts, they no longer hoped for improvement within the group and instead prioritized their own well-being. At this point, a second dimension of empowerment surfaced in a germinal form. Alongside the independent judgment that defines autonomy, participants voiced a felt obligation to protect their own health, an early expression of the responsibility that would be fully enacted only after withdrawal. In their accounts, the decision to leave grew out of the interplay of the 2.

P7 voiced this obligation in stark terms, weighing her recovery against her inability to stay away from the group.

I felt that if I went on like this, I would never get better. But as long as I stayed in the group, I could not stop myself from looking at it. So out of sight, out of mind. I had to live.

[P7, female, 25 years]

In her account, leaving was at once the conclusion of an independent assessment and an act of self-preservation. Quitting the group outright was her way of ending a pull she felt unable to resist while remaining inside it.

This concern with self-protection ran through the more specific reasons participants gave for leaving. On the one hand, individuals chose to leave to conserve their energy. Engaging with an unresponsive ISG came to feel like a waste of their time. Whether they were offering support or seeking it, the absence of meaningful feedback left their efforts feeling futile. In their accounts, such interactions consumed more energy than they provided. As a result, they decisively severed these connections to prevent further emotional depletion.

I wanted to make a difference by sharing interesting things and supporting others, but my messages were either ignored or met with polite but empty responses. Over time, this lack of engagement drained my enthusiasm. Eventually, I realized there was no point in exhausting myself on interactions that went nowhere.

[P16, female, 25 years]

On the other hand, some individuals left because they came to view the ISG as a temporary resource rather than a long-term solution. They no longer regarded the group as the primary means of addressing their challenges but rather as a tool with limited utility. When the ISG ceased to be effective or failed to resolve their problems, they chose to move on. This decision reflects a prioritization of practical problem-solving over a mere sense of belonging.

When I saw that my questions weren’t being answered, I started to feel disappointed in the group. Once I realized that real communication wasn’t possible and there was no positive feedback, I decided to leave.

[P10, female, 47 years, ISG founder]

### Courage: Overcoming the Inertia of Connection

#### Overview

Withdrawing from an ISG demanded the courage to break free from psychological inertia. In participants’ accounts, this bravery stemmed from the sense that the distress of staying in the group outweighed the anxiety of leaving. Although withdrawal may lead to feelings of loneliness and uncertainty [35], remaining in an ISG that they experienced as failing to foster healing or even worsening their condition ultimately felt more harmful. This courage manifested in 3 ways, including practicing voluntary disconnection, asserting the self through voice, and distancing from the group.

#### Practicing Voluntary Disconnection

Courage was first demonstrated through the act of disconnecting. Whether the departure was abrupt or gradual, individuals broke the habit of remaining in the group to reclaim control over their lives. They resisted the inertia of the ISG and made a deliberate decision to leave. Not every exit in the data was of this kind. P13 recalled groups from her school years that simply disappeared.

Back in middle school I joined some QQ groups. The discussions could involve suicide, so the groups often did not last long before people reported them and they were shut down.

[P13, female, 22 years]

Exits imposed in this way involved no exercise of choice, and participants did not narrate them as their own acts. The courage participants described presupposed that a meaningful choice existed to be made [10].

For some people, this disconnection was a decisive act. Once they sensed the ISG as detrimental, they severed ties without looking back to safeguard their mental well-being. In their telling, the group remained absorbed in distress while they themselves wanted to move on with life, and leaving came as a relief rather than a loss.

My decision was straightforward. I wasn’t getting what I needed, so I left without hesitation. The sense of relief after leaving was immediate.

[P2, gender unspecified, 30 years]

My mindset shifted in an instant. They just felt so irritating, and seeing the group pop up on my screen would ruin my mood. So, I quit. I want to live, but they keep talking like they want to die.

[P7, female, 25 years]

For others, disconnection was a gradual process, unfolding as they took time to weigh the pros and cons. Unlike those who left decisively, these individuals transitioned from uncertainty to clarity. Some remained in the group to actively assess its value, while others hesitated due to reliance on the ISG or fear of isolation. Whatever the reasons, the emotional cost of staying was described as outweighing any potential benefit. Once they judged that the ISG drained more than it provided, leaving followed as a considered step to prevent further harm.

When I first started feeling the group was dull, I gave it another 2 weeks to see if things would improve. Nothing changed, and I realized there was no point in letting others affect me over and over. Once I hit that limit, I left.

[P11, female, 25 years, ISG founder]

I thought about leaving many times and hesitated for nearly 3 months. My social circle was small, and I worried I’d have no one to talk to about my condition. But some members were extreme, and you could get attacked at any moment, and it really affected my mood. Considering the arguments in the group and my doctor’s advice, I decided to withdraw. I faded out slowly. At first, I felt conflicted and reluctant to let go, but that feeling eventually disappeared.

[P6, female, 26 years]

For others, disengagement could occur without the behavioral marker of exit. P15, for example, described a period during a personal crisis when her attention and emotional investment had already become completely detached from a group she still nominally belonged to before she eventually left it. P13, the only patient who never actively exited a group, described her disengagement as a quiet withdrawal of attention that followed the fading of her need for the groups.

I have probably not opened these groups for a month or 2 now. It depends on my state. When I have been doing well for a while, I do not pay attention to them at all. Even when something is posted to the group, I do not open it and just scroll past.

[P13, female, 22 years]

Muting could likewise serve as an intermediate stage rather than an endpoint. Four patients muted a group before eventually exiting it. Across these accounts, explicit exit, muting, and ceasing to check the group appeared as points on a single continuum of intentional disengagement. They differed in behavioral form and emotional intensity, but not in kind.

Whichever route participants took, the felt obligation to protect their own health that had surfaced during evaluation carried over into the act itself. P11 framed her exit as a rule of self-protection.

The moment I found the group having an emotional effect on me, I left. Living with this illness, I put my mood before everything else, and I cannot let other people affect it.

[P11, female, 25 years, ISG founder]

In her account, the obligation that had helped drive the decision to leave was already being acted on at the moment of disconnection.

For a smaller number of participants, the clarity reached at one exit did not settle. P9 described a cycle of joining and leaving that tracked the fluctuations of her condition.

I kept joining out of loneliness, wanting to find people like me, and I kept leaving out of disappointment. In different states I saw things differently. Whether I will join again, I am not sure even now. What I decide now, I may see differently after a while.

[P9, female, 36 years]

Each of her exits followed an appraisal of the group, yet the appraisals themselves shifted with her emotional state. Such accounts indicate that the courage of disconnection, and the judgments behind it, could be enacted in unstable and reversible ways.

#### Asserting the Self Through Voice

Courage is not only about leaving quietly but also about breaking the silence. For individuals prone to social withdrawal [56], speaking out against the group was a significant act of risk-taking. By voicing dissent, they stopped hiding and reasserted their boundaries, making a statement about who they are before departing.

Courage often manifested as a defense of dignity. When the ISG crossed a line, such as spreading rumors or attacking others, individuals refused to remain passive. They spoke up, not merely to vent anger, but to push back against how the group defined them. By asserting themselves, they made their departure, in their own eyes, an act of strength rather than defeat.

At first, I was very careful about what I said and tried to follow all the group’s rules, hoping to solve problems. But it seemed like no one wanted solutions. They just wanted to vent. I’m usually polite and willing to put up with personal attacks if it helps my recovery, but when someone said my illness would harm my child or that I should get a divorce, I reached my breaking point. I wrote a long message and left the group immediately.

[P4, female, 30 years]

Courage also means refusing to remain invisible. In ISGs, individual struggles often go unnoticed. Speaking out becomes a last attempt to make the group pause and recognize someone’s pain. When these heartfelt words are ignored or drowned out by arguments, staying comes to seem pointless, and speaking up becomes the final act of separation.

Sometimes, leaving is like shouting into the void. I wanted to be seen and heard, but when that didn’t happen, I knew it was time to go.

[P9, female, 36 years]

I remember someone in the group whose last message was incredibly moving. They said they felt like they were already drowning, and after being pulled out, they were just thrown into a swamp. Every punctuation mark in their post showed how desperate they were, but within seconds, their words were lost in the noise of arguments.

[C19, male, 34 years, counselor]

#### Distancing From the Group

The final aspect of courage was the ability to mentally distance oneself from the ISG, which required the strength to stand alone. This strength rests on what the empowerment framework calls self-confidence, a tool of autonomy that persisted here beyond evaluation into the moment of separation [10]. Individuals stopped identifying with the group’s collective struggle and began to prioritize their own reality over the comfort of belonging, separating their personal experience from the group’s atmosphere.

Distancing often started with validating one’s own reality in contrast to the group’s behavior. Individuals described no longer questioning the authenticity of their illness. Observing others’ exaggerated symptoms displayed for attention, they spoke of gaining clarity about their own condition and accepting their depression as a genuine issue to be managed, not a role to be performed. In their accounts, this contrast supported self-acceptance without the need for validation from the ISG.

Before this, I used to wonder if I was just acting depressed for myself. Now, after seeing how others behave, I realize they’re the ones performing, and I’ve learned to accept myself as I am.

[P12, male, 26 years]

Distancing also means perceiving personal progress by comparison. Participants described sensing that they had improved because they no longer shared the group’s deep despair. Seeing others still trapped in painful situations led them to feel they had moved forward, even if the ISG had not.

When I saw other members still struggling so much, I realized I was in a better place. I wasn’t as distressed as they were anymore.

[P17, female, 24 years]

Finally, distancing means letting go of an environment that holds them back. Individuals came to see ISGs not as a source of support but as a burden. Staying in the group felt like sinking deeper into the same problems, so leaving becomes a necessary step toward moving on and embracing a new life.

I feel like I need a fresh start. My old life and social circles were like a swamp, always dragging me down. Social connections matter, and it’s important to move upward. Leaving the group was my way of saying goodbye to that old life.

[P7, female, 25 years]

### Responsibility: Managing the Recovery Process

#### Overview

Responsibility, in this context, refers to the transition from dependence on others to active management of one’s own health. This dimension did not first appear after exit. The felt obligation to protect one’s own health had helped drive the decision to leave and was already being acted on at the moment of disconnection. After withdrawal, that obligation matured into the sustained management of one’s own recovery. While some participants described having achieved full recovery, many remained in the process of healing. They came to feel that passively waiting for comfort from external sources was ineffective and that progress required proactive effort. In the empowerment framework, such responsibility encompasses developing goals, working with providers and others, taking on decision-making tasks, engaging in self-care, and living with the consequences of one’s choices [10].

Living with those consequences often came first. Leaving the group removed a familiar source of relief, and some participants described bearing the loneliness and uncertainty that followed.

After leaving, I worried I would have no one to talk to about my condition. And I no longer message people privately. After everything that happened, I do not want to build relationships with strangers anymore.

[P6, female, 26 years]

Against this background, the journey toward responsibility unfolded across 3 themes. Participants redirected their personal energy, refined their avenues for recovery, and internalized recovery as a felt obligation.

#### Redirecting Personal Energy

The first step in assuming responsibility was to redirect one’s energy consciously. Rather than expending effort on the ISG, participants began to invest in their own lives. They shifted their focus from the distractions of the online environment to tangible actions in daily life. In their accounts, dwelling on group dynamics had drained their mental resources without yielding meaningful change. They therefore turned their attention to daily routines and practical tasks, where they felt able to exert genuine control.

I no longer concern myself with those issues. Now, my thoughts revolve around what I’ll eat tomorrow, where I’ll take my child for fun, and how I can improve at work.

[P7, female, 25 years]

Since leaving, I’ve become more attuned to my immediate environment and community. Living authentically and engaging with reality has gradually become my focus, and it’s made a real difference for me.

[P2, gender unspecified, 30 years]

Additionally, individuals channel their energy toward sources of constructive support. Taking responsibility means actively seeking out environments and relationships that foster growth, rather than perpetuate negativity. The emotional resources freed up by leaving the ISG are invested in new hobbies, meaningful connections, and activities that promote well-being.

The negativity in the ISG was overwhelming. After leaving, I started pursuing things that genuinely interest me and bring positive energy. I feel like I’m moving forward. Now, I surround myself with people and activities that uplift me, creating a cleaner, more supportive environment.

[P11, female, 25 years, ISG founder]

#### Refining Avenues for Recovery

Taking responsibility also meant curating a support system tailored to personal needs rather than relying on the unpredictable interactions of large ISGs. Individuals learn to prioritize safety and stability, often gravitating toward smaller, interest-based communities or even nonhuman forms of support, such as AI agents. In participants’ accounts, what these alternatives had in common was the absence of the volatility and judgment they had encountered in larger groups.

The bigger the group, the more exposed you are to criticism and conflict. For me, ISGs actually increased my risk of relapses. I realized I am better off avoiding those environments, so I joined smaller, hobby-focused groups where discussions stay positive and relevant.

[P1, male, 48 years]

Over the past year, ChatGPT has been the most helpful companion. I can share anything without fear of judgment. It offers emotional support and psychological insight, something I couldn’t find with people.

[P9, female, 36 years]

Furthermore, responsibility entailed acknowledging the physiological aspects of depression and seeking appropriate medical intervention. In their view, emotional support alone could not address the physiological underpinnings of their condition. Combining professional treatment with self-education was their way of working toward lasting recovery rather than temporary comfort.

After leaving the group, I began medical treatment. The side effects were tough. I spent most days just sleeping, but medical care was essential. Severe depression is fundamentally a physiological issue, and medication helps restore my sleep and physical health. Reading and reflecting helped me understand my body and how to get better. Medical care and learning truly helped me out of that dark place.

[P10, female, 47 years, ISG founder]

For some participants, recovery was to be found not in conversation but in purposeful action. When neither peer support nor counseling brought relief, they turned to concrete goals, such as advancing their education or career, as a means of managing distress. In their accounts, achievement and progress became therapeutic, offering a sense of control and accomplishment.

I tried counseling, but it felt mechanical and only offered emotional comfort. What I really needed was to solve my problems. So, I decided to prepare for my postgraduate exams. It was still painful, but it helped by shifting my focus away from my internal struggles. I had a larger conflict to overshadow my smaller one, even though that smaller conflict remains.

[P15, female, 27 years]

#### Internalizing Recovery as a Felt Obligation

Ultimately, responsibility meant internalizing recovery as a felt obligation, one recognized from within rather than imposed from outside. Individuals described no longer relying on the ISG for solutions and instead taking ownership of their healing process. In this shift, recovery was no longer a passive hope but an active commitment sustained by planning, self-care, and follow-through.

Some participants talked about how they learned to plan their own treatment, moving from vague discussions to concrete steps. Because ISGs often lack professional guidance, participants described having to navigate the complexities of medical care on their own.

The ISG did mention seeing a doctor, which I hadn’t considered before joining. But it didn’t offer real guidance, like which doctor to see, how to make appointments, or what precautions to take. Every new patient needs this information, but I had to figure it out myself, step by step, because the group just did not provide those details.

[P10, female, 47 years, ISG founder]

Others emphasized the importance of self-care and daily discipline. Rather than surrendering to helplessness or rumination, they focus on practical routines and personal growth. Taking action, no matter how small, becomes their way of maintaining stability and fostering resilience.

Stay active in your life. Don’t let yourself become idle, because that leads to overthinking and inaction.

[P18, female, 38 years, ISG administrator]

I read a lot, mainly psychology, philosophy, and biographies, and I just do what needs to be done. As I get older, my perspective keeps evolving, and I see that as a subtle but meaningful improvement.

[P2, gender unspecified, 30 years]

Finally, some participants learned to derive their sense of worth from within, rather than seeking validation or security from the group. Having come to feel that depending on others for stability was precarious, they cultivate an internal foundation for self-esteem and well-being.

I stopped looking for support from others. I don’t need a group to feel secure or recognized anymore. I can give myself that sense of value.

[P16, female, 25 years]

Not all participants, however, attributed this rebuilding to the exit itself. P10, whose treatment and self-education appear above, was explicit that her recovery had proceeded on its own track.

My mood was stabilized by medication, and life went on steadily under my own arrangements. Treating depression is a very long process. I spent 2 or 3 years in treatment and another 3 or 4 in relapses, and it is hard to say whether the changes in that period were positive or not. But none of this had anything to do with that group.

[P10, female, 47 years, ISG founder]

Her experience marked the limit of what can be attributed to withdrawal itself. Leaving could clear the space in which treatment and self-directed effort proceeded, but in accounts like hers, these efforts, not the exit, accounted for the recovery.

## Discussion

### Principal Findings

#### Central Aim

This study investigates the lived experiences of individuals with depression who choose to withdraw from ISGs. While previous research often interprets member attrition as a sign of intervention failure or waning motivation for mutual aid [19,25,57], our findings offer a different perspective. Withdrawal is not necessarily the termination of empowerment but can be an active strategic process. We conceptualize this as strategic withdrawal, a journey shaped by autonomy, courage, and responsibility. Through this process, participants disengage from dysfunctional digital connections and reclaim agency in their recovery. In the context of digital health, empowerment is thus not only about forming connections [3,11] but also about exercising the autonomy to disconnect when necessary.

#### Deconstructing Strategic Withdrawal: Autonomy, Courage, and Responsibility

The process of strategic withdrawal begins with a realistic reassessment of the ISG. Unlike studies that focus on the benefits of community support [3,11,12], this study shows that empowerment often arises when individuals come to perceive the group as limited or even harmful. This shift from idealized expectations to critical evaluation allowed participants to conserve their energy. Their accounts traced a movement from passive recipients to agents who made decisions based on their own well-being.

Leaving the group is also an act of courage. Whether the departure is abrupt or gradual, individuals break free from the inertia of habitual connection. Decisive exits establish boundaries through immediate action, while more measured withdrawals allow individuals to test their limits and reduce anxiety through rational trial. Both approaches demonstrate that empowerment involves the willingness to step outside familiar routines and reclaim control over personal boundaries.

Responsibility, finally, surfaced during evaluation as a felt obligation to protect one’s own health. This obligation was itself part of what drove the decision to leave. After withdrawal, responsibility matured into enacted self-management. Participants redirected their energy toward constructive activities and alternative forms of support, including offline pursuits and support from AI agents. They also came to treat recovery as their own undertaking. The movement here is not from one dimension to the next but from a germinal to a mature form within the same dimension.

Taken together, the 3 dimensions operated jointly rather than separately. Separation imposed from outside, with no choice exercised, was not experienced as empowering. Critical judgment alone did not amount to withdrawal until courage carried it into action. And responsibility was already present, in germinal form, in the very decision that autonomy grounded. Withdrawal took on its empowering character through this staggered unfolding and interplay across the process, rather than through any single dimension.

It is important to note that withdrawal is not without its challenges. Individuals must navigate the social isolation and uncertainty that can follow detachment. Nonetheless, many participants confronted these difficulties rather than remaining in a sense of belonging they no longer experienced as genuine. In their accounts, this choice reflected their maturation as active managers of their own recovery.

These claims are subject to limits that our negative cases made visible. Exits imposed from outside involved no exercise of choice. Some participants cycled between joining and leaving as their condition fluctuated, and their judgments proved provisional and reversible. Others attributed their recovery to treatment and self-directed effort and regarded the exit itself as inconsequential. Strategic withdrawal thus designates a particular kind of leaving rather than all forms of leaving, and the empowerment it carries should not be read as a property of attrition in general.

#### Empowerment

Our findings challenge the retention-focused paradigm that has dominated ISG research. Traditionally, empowerment has been equated with ongoing participation and frequent interaction [19,24], leading to the assumption that attrition signals failure [21,25,57]. However, this perspective overlooks the risk of digital dependency, where constant connectivity can undermine autonomy [10]. We argue that strategic withdrawal is not a setback but a corrective action. It is an exercise of autonomy and self-regulation when the group environment no longer supports recovery.

Strategic withdrawal also embodies courage. While previous studies highlight the role of anonymity and group empathy in fostering self-disclosure [3,11], our results suggest that leaving the comfort zone of an ISG requires equal, if not greater, bravery. Individuals must overcome the fear of loneliness and break free from the inertia of collective dependency to protect their personal boundaries [10]. In digital spaces rife with emotional contagion, empowerment is reflected not only in the ability to connect but also in the capacity to disconnect when necessary [37,38].

Finally, strategic withdrawal enacts a responsibility that had already begun to form before the exit. The felt obligation to protect one’s own health, which helped drive the decision to leave, matured after withdrawal into active self-management [10]. ISGs can serve as practice grounds for reintegration into real life [3], and those with greater motivation for recovery may choose to exit earlier [3]. In this sense, withdrawal is not necessarily a sign of failure but can signal progress: the transition from “digital patient” to an autonomous, self-sufficient individual.

### Findings in Context

#### Dynamic Engagement Under Depressive Fluctuations

Given the episodic and fluctuating nature of depression, individuals’ involvement in ISGs tends to evolve and fluctuate over time [3]. Building on prior research that highlights variations in participation styles [3,41,42], our study broadens this perspective by examining fluctuations not only in engagement but also in the processes of connection and disconnection. While previous research has focused on retention factors [19,24,25,57] and often portrayed participants as passive actors shaped by network positioning [13,21], our study reveals that individuals are capable of strategic, independent decision-making.

This perspective reframes high attrition rates in ISGs [19]. Rather than signaling intervention failure, attrition can be seen as a purposeful reallocation of energy in response to changing needs. Individuals do not simply wait for support; instead, they actively assess and adjust their resource use [3,58]. Withdrawal, like lurking or active posting, is a deliberate choice tailored to recovery goals [3]. The cyclical accounts in our data illustrate that withdrawal may function as a periodic adjustment rather than a permanent break. This recognition underscores that even disconnection can be part of an active, self-directed recovery process.

#### Boundary Management Under Stress

Our findings corroborate prior research on the pressures of constant online engagement and the risks of emotional contagion in ISGs [31-33]. When negative group dynamics lead to empathy fatigue, withdrawal becomes a vital strategy for managing digital boundaries [59]. What was once a sanctuary can become a source of emotional exhaustion [11,60], prompting individuals to sever ties and protect their energy.

More importantly, this act of boundary-setting is not an escape but a deliberate reconstruction of control. When individuals come to see the ISG as perpetuating a patient identity rather than supporting recovery, withdrawal becomes a means of self-governance [61]. The subsequent shift to AI-powered support or offline activities is not merely a change of environment but a move toward interactions that participants experienced as safer and more manageable. By choosing low-pressure interactions, individuals avoid information overload and unmet needs [3,11,30], reclaiming a sense of autonomy within more manageable boundaries [6].

### Practical Implications of ISG Use

For mental health professionals, it is crucial to recognize the temporary and transitional nature of ISGs. While these groups are often recommended as supplements to traditional treatment [3], such recommendations should be time-limited and clearly framed. Group administrators can support this process by clearly stating, at the point of entry, what the group does and does not offer. For instance, a pinned group notice could specify that the group provides peer-based mutual support rather than professional treatment. It could also outline the kinds of needs the group is and is not equipped to address. Establishing this shared understanding from the outset may help prevent the disappointment that arises when professional guidance is expected but not available. Professionals can help patients view ISGs as practice grounds for recovery, emphasizing that withdrawal is a healthy and expected step when the group environment becomes distressing or unhelpful. This approach can alleviate feelings of guilt or anxiety associated with leaving, facilitating a smoother transition from online support to offline management.

Some participants described turning to unstructured resources such as ChatGPT and similar AI-based tools for emotional support after withdrawal. This reflects a real and growing practice. However, such use should not be regarded as equivalent to professional care or validated peer support, given ongoing concerns about safety, accuracy, and lack of clinical oversight. Professionals should therefore ask about and remain attentive to patients’ use of these resources after leaving an ISG.

For participants, developing digital boundary management skills may be important. To support this, professionals should look beyond activity metrics such as posting frequency or response rates and encourage individuals to monitor their ISG experiences for signs of stress. When a negative environment begins to erode psychological control, participants can proactively adjust their engagement [58], for example, by shifting from high-pressure ISGs to lower-pressure tools or routine activities to enhance resilience.

Finally, the evaluation of ISGs should move beyond retention rates and focus on recovery agency. Continuous participation does not always signal successful empowerment. Instead, a successful ISG should be measured by its ability of the group to foster self-efficacy and support independent health management [6,7]. Leaving the group to pursue autonomous recovery should be recognized as a positive outcome, not a failure. Expanding these metrics will help build a more resilient and patient-centered digital support ecosystem.

### Limitations

This study has several limitations. First, although our sample included individuals who have withdrawn from ISGs, our findings are based on retrospective self-reports. Participants’ recollections of their withdrawal experiences may be shaped by their current psychological state, potentially leading to reinterpretations or selective memory [62,63]. Second, despite our efforts to recruit a diverse participant pool, selection biases may remain. For example, the perspectives of those who experienced profound isolation or who completely severed digital ties after leaving may not be fully represented. Third, while a few participants were members of professionally managed ISGs, most belonged to informal, privately organized groups. These differ substantially from regulated commercial communities with formal oversight, so our conclusions may not be generalizable to all types of ISGs.

### Future Research

Future research could adopt longitudinal designs to track recovery trajectories over extended periods. For example, comparing the social and psychological outcomes of strategic versus passive leavers after 12-24 months could clarify the long-term effects of withdrawal. In addition, mixed methods studies leveraging big data could help identify behavioral indicators that signal when individuals are approaching their limits. Such insights would enable platforms to offer timely support or interventions, helping users make informed decisions about group participation before reaching a point of burnout.

### Conclusions

For many individuals with depression, leaving an ISG is not simply the end of support. It may mark an empowering milestone in their recovery journey. By examining the mechanisms underlying attrition, this study identifies strategic withdrawal as a process in which participants actively manage their online boundaries, regulate emotional energy, and prioritize personal recovery needs. Empowerment in this context is multidimensional and dynamic. Autonomy grounded the decision to leave and persisted as participants judged their recovery by their own standards. Courage carried that judgment into the act of disconnection against the pull of habitual connection. Responsibility surfaced before the exit as a felt obligation to protect participants’ own health and matured afterward into the daily work of self-management. It was in this staggered unfolding and interplay of the 3, rather than in any one of them, that withdrawal took on its empowering character.

The true value of ISGs lies not only in fostering connection but also in serving as transitional spaces where users can exercise their right to disconnect. Our findings suggest that attrition in digital mental health should not be viewed solely as a failure. When withdrawal is a strategic choice for self-regulation, it represents a meaningful form of empowerment. We recommend that health professionals and platform developers recognize and support this process as a legitimate pathway for individuals to move from virtual dependency toward autonomous recovery.

## Supplementary material

10.2196/92690 Multimedia Appendix 1 Interview guideline.

## Abbreviations

ISG: internet support group
