# Supplementary material for: Strategic Withdrawal as Empowerment for Individuals With Depression in Internet Support Groups: Qualitative Study
Source: J Med Internet Res. 2026 Aug 14;28:e92690. doi: 10.2196/92690 (PMC13475938; doi:10.2196/92690)
Supplement: Supplementary file 1 [file jmir-v28-e92690-s001.docx]

**Interview guideline:**

By: Yufan Zhang and Zhentao Liang

I. **Basic information**
1. Could you tell me your gender, occupation, educational background, and age?
2. When were you first diagnosed with depression?
3. When did you join the ISG? How long did you stay, and when did you leave?
4. How would you describe your emotional state or psychological difficulties before joining the ISG?
5. Had you tried other forms of help at that time (e.g., offline counseling, talking with family or friends)?
II. **Motivations for joining the ISG**
1. How did you first learn about the ISG, and what motivated you to join?
2. Compared with other forms of support, what made the ISG particularly appealing to you?
3. At the beginning, what types of help or responses were you hoping to receive?
4. What were your first impressions of the ISG when you joined?
III. **Experiences during participation in the ISG**
1. What was your usual style of participating? Did you actively post and interact, or did you mostly browse?
2. Did you ever feel particularly recognized, supported, or cared for within the ISG? Could you describe those moments?
3. Were there experiences that made you feel uncomfortable, troubled, or affected by a negative atmosphere? What contributed to those feelings?
4. Have you encountered situations such as being ignored after posting, feeling compelled to remain silent, or having conflicts with other members?
5. Overall, would you say the ISG had a more positive or negative impact on your emotions? Did this change over time?
IV. **Process of leaving**
1. Under what circumstances did you begin thinking about leaving the ISG? What was on your mind at that time?
2. Were there specific events, content, or emotional triggers that prompted your decision? Did you struggle or hesitate before leaving?
3. How did the withdrawal process unfold for you?
4. Did you experience mixed feelings, such as guilt, regret, or relief, when leaving?
5. In your view, what does leaving the ISG mean to you?
V. **Changes and actions after leaving**
1. After leaving the ISG, did you seek other sources of support (e.g., offline counseling, talking with family or friends)? If so, were they helpful?
2. Since leaving, have there been any changes in your emotional state, daily routine, or social interactions? Would you describe these changes as positive, negative, or neutral?
3. Have you considered rejoining an ISG? Why or why not?
4. Is there anything else you would like to share that was not covered in this interview but you consider important?
